# Phenol disgrace via Periodate in integrating by using Supersonic Radiation

**Published:** 2015

**Authors:** A Seid-Mohamadi, G Asgari, R Shokoohi, S Adabi

**Affiliations:** * Social Determinants of Health Research Center, Department of Environmental Health Engineering, School of Public Health, Hamadan University of Medical Sciences, Hamedan, Iran,; ** Department of Environmental Health Engineering, School of Public Health, Hamadan University of Medical Sciences, Hamadan, Iran

## Abstract

In this study, a successful degradation of phenol was achieved by a combination of processes, ultrasonic irradiation and periodate. The effect of pH, dosage of IO4-, dosage of initial phenol and ultrasonic irradiation time on the phenol degradation were examined. Furthermore, the impacts of ion intensity on phenol degradation were examined. The findings indicated that the disgrace ratio advanced in acidic conditions and an upper degradation was achieved in combination processes. The current new investigation examined the effect of ion intensity and the findings determined that the principal intensity of solution is an inactive variable on phenol disgrace with these systems. A comparison research among IO4-/ US system and IO4- and US separately determined the COD removal and indicated that an combined method of IO4-/ US system had the best execution.

## Introduction

Industries generate a considerable amount of polluted wastewater and they have recently produced a different of concerning problems in the aqueous resolutions as a result of inadequate treatment operations [**1**]. Organic compounds have been applied widely in the construction manners. The presence of organic pollutants in aquatic resolutions is one of the major social and environmental awareness. Groundwater and surface waters are vulnerable to contamination by industrial wastewaters. Scientists have been concerned about the nature of natural and original parts and their harmful effect in water sources. Therefore, governments have authorized hard and fast laws to protect the environment in the last decades [**2**]. 

Phenol, one of the usual parts, has been consumed in several applications like pesticides, paint, and stain applications, natural chemicals production, pharmaceuticals. As a consequence, it realized in the effluence of these industries [**3**]. The impact of this organic compound was investigated in aqueous solutions and based on the EPA declaration, it is a priority pollutant. Accordingly, the standard phenol focouses in the effluent stream is less than 1 ppm [**4**]. According to the recent research, many methods have been studied to destroy phenol residuals in the aqueous solutions albeit most of them have a variety of limitations. Therefore, it is important to find an efficient process to remove or degrade phenol residuals before the discharge to water sources. Advanced Oxidation Processes (AOPs) have been studied as efficient methods in last decades [**5**,**6**]. Methods like Fenton, radiolysis, photo catalytic oxidation, sonication, periodate oxidation, ozonation, etc., are expedient because of their potential to generate hydroxyl radicals in aqueous solutions [**7**-**9**]. Of these methods, the Ultrasonic method is a vital approaches because it has a low cost and it is comfort to handle. The basis of ultrasonic reaction is on hydroxyl generations that are generated via reactor and related to the power of the reactor that makes numerous frequencies [**10**]. The aquatic cavity bubbles have been grown and impulse collapsed all molecules of gases and water vapour from the aqueous similarly, the variety of radicals being generated according to Eq1 to Eq3 [**11**]. 

H2O+))) → OHo + Ho (1)

O2+ ))) →2O0 (2)

OHo + Oo → OO (3)

These phenomena caused the degeneration of natural composites such as phenol in aqueous solutions. The degradation proceeds mainly by two reaction mechanisms: direct pyrolysis in and around the collapsing bubbles, and oxidation by OHo radicals [**12**]. Whether, phenol is the stable organic compounds, the ratio of sonodegradation of that is obviously low. There are some reports in recent studies that imply the combination processes like periodate oxidation to achieve the aim [**13**]. 

Iodine oxide anionic sorts created by the bonding of the iodine molecule with a various amount of oxygen molecules are the hypoiodous anion (IO−), the IO3−, the periodate anion (IO4−), the ms periodic anion (IO53−) and the role cyclic anion (IO62−). The full firm cases in their acids and spices are the periodate and part recurrent anions. The periodate ion is available in sodium salt (readily soluble) and potassium salt of limited solubility [**14**]. The periodate anion acts via different synthetic composites like natural mixtures. The oxidation effect pathway related to the chemical response parameters [**15**].

Periodate has a good power to oxidize the organic mixtures, particularly in the closeness of hydroxyl, once this substance is activated, it changes into stronger radicals such as IO40 and IO30. These radicals were produced in two stages: in the initial level, one of the chemical bands IO was attacked to one of the chemical bonds OH, and next in the second level, the chemical bonds have formed a ring [**16**]. For these reasons, the combination of sodium periodate with ultrasonic process have more power to remove the organic compounds such as phenol in aquatic solutions.

Saidmohammadi et al. [**17**] investigated the disgrace of 2,4 dichlorophenolindophenol by periodate, persulfate and hydrogen peroxide in the proximity of US. Others prophesied that in the proximity of US, periodate is a good catalyst which enhanced the degradation.

Rashmi et al. [**10**] investigated the phenol degradation with ultrasonic reactors and via peroxide of hydrogen, zero valent metals and ozone. Studies clearly showed that the phenol degeneration is increased the closeness of the reactant.

In the present research, the enhancement in the rates of ultrasonic phenol degeneration in the aqueous resolution via sodium periodate, to activate periodate, was studied. Eventually, the oxidations kinetics in all systems was analyzed. Phenol removals in the effects circumscribed via utilizing the Spectrophotometer. The COD test emplyed for the phenol investigation.

## Experiment

**Materials **

Phenol (163 g/ mol) was obtained from MERCK, sodium periodate from MERCK, potassium Ferrocyanide (K3Fe(CN)6) and all the other regents were obtained from MERK and utilized as received. The analytical reagents grade or better chemicals as well as Milli-Q water utilized in the test.

**Apparatus**

**Fig. 1:**
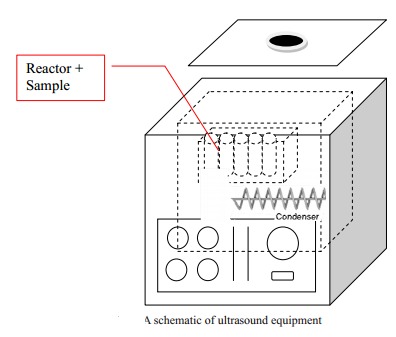
Ultrasonic equipment

The next pieces of equipment were employed in the research:

1. Ultrasonic equipment: LUC405 model, range of temperature 0 to 50oC, made in Korea. The process illustrated in **Fig. 1**.

2. Spectrophotometer: model Uv/ Vis, made by Perkin Elmer.

**Us/ IO4 system**

Batch tests conducted in a rotary shaker at 25 ċ and 125 rpm. The s tock solution of phenol 1000 mg/ l and priodate (213.89g/ mol) are produced in deionized water before to each plate test. The pH amounts of all solutions are set via (0.5N) sodium hydronized (NaOH) or H2SO4 95%. Multiple sets of tests performed to ascertain the results of different factors on phenol decay. To evaluate the impact of time on the phenol decay, it was studied at every 15 min, from 15 min to 120 min. To survey the impact of pH on the phenol decay, three pH regimes of aqueous solution at 3.0, 7.0, and 11.0 investigated. In the other tests, the optimized pH tuned. To evaluate the impact of IO4 on phenol degradation, from 1 to 7 mMIO4 in the specific phenol concentration (50 mg/ l) and to evaluate the impact of the initial phenol on this degradation skirt, 25 to 200 mg/ l phenol were used. To survey the impact of ion on phenol degradation, calcium chloride amounts of 1.03, 0.52, and 0.13 utilized. To evaluate the impact of ultrasound, the resolution irradiated via ultrasound for 120 minute. 

**Analytical techniques**

Phenol removals in the reactions were determined by using the Spectrophotometer at 500 nm. COD tests were furthered utilized for the phenol examination [**18**].

## Results 

**pH effect**

**Fig. 2:**
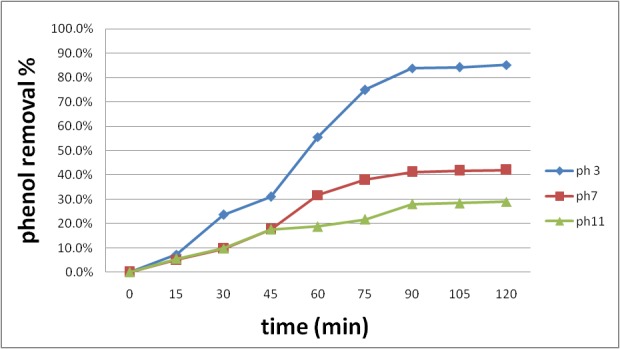
Effect of pH on the phenol disgrace by IO4-/ US system. IO4- 5mM, phenol 50 mg/ l

In all the chemical reactions, pH is one of the major factors that directly affect the whole chemical operation [**19**]. Therefore, a sort of tests modeled to survey the impact of pH on phenol degradation in IO4-/ US system. Experiments conducted at a pHs of 3.0, 7.0, and 11.0. The findings depicted in **Fig. 2**, and showed that the phenol degradation was obviously increased at a 3 pH and findings indicated that the efficiency was of 85.20% after 120 min, nevertheless after 90 min the phenol disgrace efficiency was almost constant and at 90 min, the efficiency was 83.80%.

This fact happening due to hydroxyl radicals was caused by the generation by ultrasound in an acidic condition; subsequently more IO4 and IO3 generated Eq4 to Eq 7 [**20**].

2IO40 →2IO8 (4)

2IO5+H2O→IO3- +IO4- +2H+ +O2 (5)

2IO30→2IO6 (6)

2IO6 + H2O →IO3- +IO4- +2H+ (7)

**Effect of periodate concentration**

**Fig. 3:**
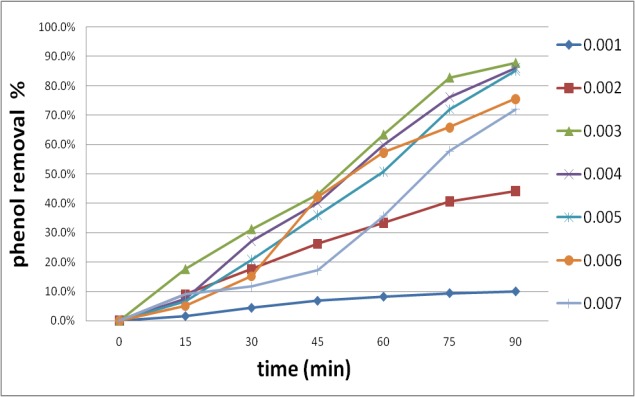
The influnce of periodate concentration mM on the phenol disgrace by IO4-/ US system. pH = 3, phenol 50 mg/ l, time 90 min

Tests carried out to specify the most efficient periodate dosage for the phenol disgrace in the ultrasound waves existence. The periodate focuses of 1-7 mM were applied. The results are depicted in **Fig. 3** and announced that phenol degradation efficiency was of 10%, 87.80%, 72% with IO4- ; 1, 3 and 7 mM respectively after 90 min. The phenol degradation efficiency was low at little periodate concentrations as if at upper focuses. These results can be expressed that periodate directly reacts with the hydroxyl in the solution, in low dosage less periodate reacts with hydroxyl; instead in high dosage, hydroxyl engages in the interfering reactions of Eq. 8 [**21**].

OH0 + IO4- → OH- + IO40 (8)

**Impact of first concentration of phenol**

**Fig. 4:**
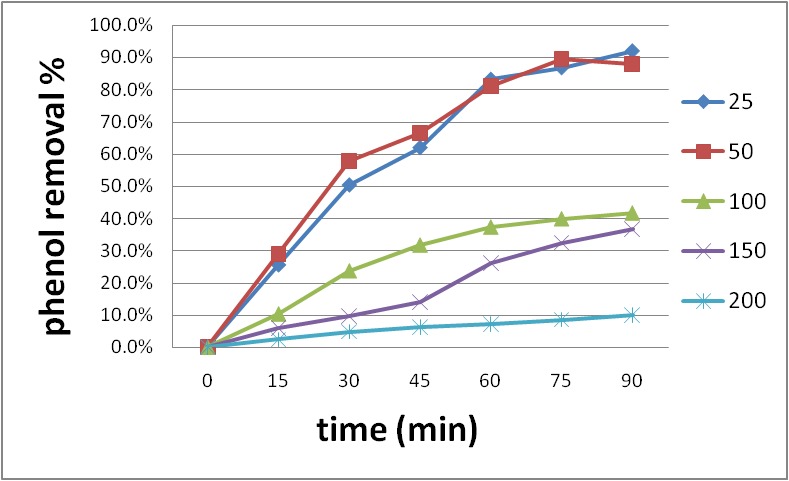
Impact of first phenol focuses on the phenol disgrace by IO4-/ US system. pH =3, IO4- 3mM, time 90 min

The study continued under an identical IO4- concentration at an optimized pH was carried out in the same ultrasonic frequency at an initial phenol of 25, 50, 100, 150, and 200 mg/ l. During sonication, the efficiency was 92, 87.8, 41.6, 36.6 and 10 after 90 min, as shown in **Fig. 4**. With the mount up of the phenol concentrations, the efficiency decreased, whose reaction needed more periodate dosages and hydroxyl radicals [**22**].

**Effect of ultrasonic and peridate solely**

**Fig. 5:**
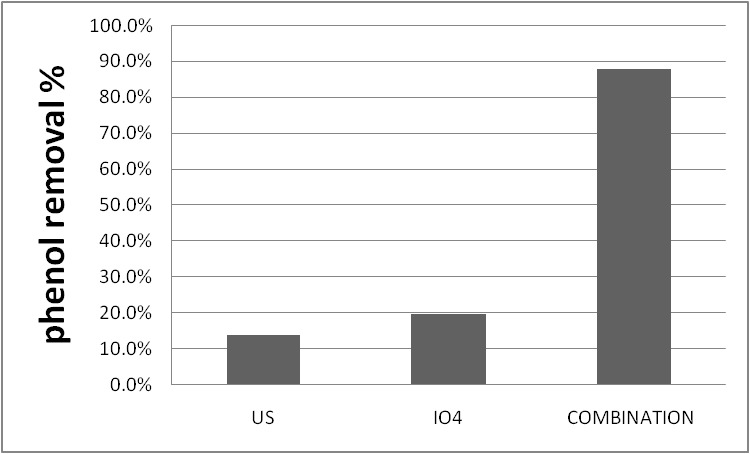
Effect of ultrasonic and peridate solely and the combination on phenol disgrace. pH =3, phenol 50 mg/ l, IO4- 3mM, time 90 min

Firstly, the ratio of phenol disgrace was investigated by using just ultrasonic at 50 mg/ l concentration of the phenol and the received findings were presented in **Fig. 5**. As the figure illustrates, the rate of degradation was 14% after 90 min. Secondly, the phenol degradation rate was investigated just in presence of peridate of 3 mM at 50 mg/ l concentration of phenol, and the results were shown in **Fig. 5**; the rate of degradation was 19.6%. These experiments showed that the application of sonication and periodate alone is not capable of a full degradation of the phenol. Therefore, in order to catch higher removal efficiency, the combination of the oxidants and ultrasonic must be applied [**23**]. 

**Effect of ion intensity on the degradation of phenol by IO4-/ US system**

For this system, three sets of tests conducted in the present investigation. In the current instance, 0.13, 0.5, and 1.03 g/ l of CaCl were used and served as additions to aqueous solutions. The study continued under identical IO4- concentrations at optimized pH and was conducted in the same ultrasonic frequency at 90 min for this system at initial phenol concentrations of 50 mg/ l.

**Fig. 6:**
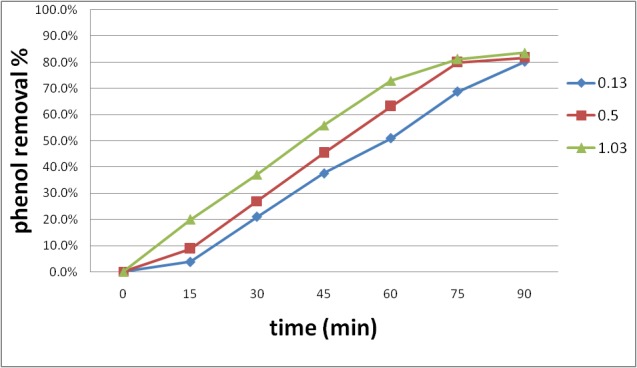
The impact of ion intensity on the decay of phenol by IO4-/ US system. pH =3, phenol 50 mg/ l, IO4- 5mM, time 90 min

The findings presented in **Fig. 6** indicated that the phenol degradations were obviously increased at 1.03 g/ l CaCl, as related to 0.13 g/ l CaCl. This can be expressed via the ion effect created by CaCl, hence this caused the movement of phenol molecules to the interaction of the cavities made via the sonication [**3**]. Nevertheless, the maximum rate of phenol degradation in presence of CaCl in IO4-/ US was 83.6%, as related to the maximum ratio of phenol decay in the lack of CaCl; in IO4-/ US it was 87.8%, provided that the ion intensity of solution is an inactive variable in both systems in phenol degradation.

## Conclusions 

This study showed that the application of ultrasonic frequency with periodate, separately degraded phenol in aqueous solutions. Nevertheless, the ratio of phenol disgrace in combination with systems like IO4-/ US are more than separate systems. The study also showed that the system required an acidic pH for effective phenol removal and the phenol degradation enhanced in low concentration of the initial phenol. This study also showed that COD reduction was decreased since time pass. Moreover, IO4- was an effective substance that enhanced the phenol degradation in IO4-/ US system. Furthermore, the study showed that the application of comfort additions like CaCl did not have any obviously positive effect on the phenol degradation. 

**Acknowledgments**

The authors appreciate the support of Hamadan University of Medical Science, the Faculty of Health. 
